# High-Performance Liquid Chromatography–Fluorescence Detection Method for Ochratoxin A Quantification in Small Mice Sample Volumes: Versatile Application across Diverse Matrices Relevant for Neurodegeneration Research

**DOI:** 10.3390/toxins16050213

**Published:** 2024-05-03

**Authors:** Elba Beraza, Maria Serrano-Civantos, Maria Izco, Lydia Alvarez-Erviti, Elena Gonzalez-Peñas, Ariane Vettorazzi

**Affiliations:** 1 MITOX Research Group, Department of Pharmaceutical Sciences, School of Pharmacy and Nutrition, Universidad de Navarra, 31008 Pamplona, Spain; eberaza@alumni.unav.es (E.B.); mserrano.14@alumni.unav.es (M.S.-C.); mgpenas@unav.es (E.G.-P.); 2 Laboratory of Molecular Neurobiology, Center for Biomedical Research of La Rioja (CIBIR), Piqueras 98, 26006 Logroño, Spain; mizco@riojasalud.es (M.I.); laerviti@riojasalud.es (L.A.-E.)

**Keywords:** ochratoxin A, HPLC-FLD, tissues, brain, plasma, mouse

## Abstract

Ochratoxin A (OTA) is a mycotoxin commonly found in various food products, which poses potential health risks to humans and animals. Recently, more attention has been directed towards its potential neurodegenerative effects. However, there are currently no fully validated HPLC analytical methods established for its quantification in mice, the primary animal model in this field, that include pivotal tissues in this area of research, such as the intestine and brain. To address this gap, we developed and validated a highly sensitive, rapid, and simple method using HPLC-FLD for OTA determination in mice tissues (kidney, liver, brain, and intestine) as well as plasma samples. The method was rigorously validated for selectivity, linearity, accuracy, precision, recovery, dilution integrity, carry-over effect, stability, and robustness, meeting the validation criteria outlined by FDA and EMA guidelines. Furthermore, the described method enables the quantification of OTA in each individual sample using minimal tissue mass while maintaining excellent recovery values. The applicability of the method was demonstrated in a repeated low-dose OTA study in Balb/c mice, which, together with the inclusion of relevant and less common tissues in the validation process, underscore its suitability for neurodegeneration-related research.

## 1. Introduction

Ochratoxin A (OTA) is a mycotoxin produced naturally as a secondary metabolite by some species of *Aspergillus* and *Penicillium* fungi that can contaminate a great variety of foods, such as cereals, beans, coffee nuts or species [1]. It is a thermostable compound that is not destroyed by common food preparation procedures and therefore enters the food chain via both raw and processed products [1]. In this regard, several human biomonitoring studies have demonstrated human exposure to this mycotoxin [2].

OTA is a potent renal carcinogen in rodents, which has been proven to cause renal tumors in rats and mice, with male rats being the most sensitive [1,3,4,5]. Considering this, but without having enough evidence in humans, the International Agency for Research on Cancer (IARC) classified it in 1993 as a possible human carcinogen (group 2B) [6], and maximum levels based on this effect have been established in several food commodities to reduce health risks in Europe [1]. However, even though its target organ is the kidney, OTA has also been described as immunotoxic, hepatotoxic, teratogenic, and neurotoxic [7,8,9,10,11,12]. 

In recent years, research has examined the impact of both acute and chronic exposure to OTA on the nervous system, revealing neurotoxicity as a sensitive endpoint for this mycotoxin [13,14]. In vivo, OTA has been proven to alter the proliferation and differentiation of cells in the hippocampus [15] and in the subventricular zone of adult mice [14]. Moreover, investigations have revealed the possibility of OTA causing parkinsonism in mice, demonstrating that the nigrostriatal pathway might be affected by OTA [7,16,17,18]. The relevance of these findings underlines the importance of carrying out studies involving OTA administration in mouse models, given that mice currently stand as the most widely used experimental model in neurodegenerative disease research. In order to further delve into this matter, it is crucial to develop methodologies able to determine OTA in biological samples of mice. In addition, in the context of neurodegenerative diseases, and more specifically Parkinson’s disease (PD), it is crucial to quantify OTA not only in plasma, kidney, and liver, which are the most common organs reported in the literature when studying OTA, but also in brain and intestine tissue. The relevance of intestine tissue stems from Braak’s hypothesis, which proposes that PD might initiate at the enteric nervous system and spread to the lower brainstem via the vagal nerve [19].

There are methods reported in the literature to measure OTA in biological samples, but they have either been developed for other species, use large sample volumes, do not include certain relevant tissues, or lack validation data. Indeed, considering that rats are the most sensitive to the toxin, most of the methods developed to quantify OTA for toxicology studies use rat biological samples [20], and adapting them for their use with mice presents unique challenges. An analytical method for OTA quantification in mouse samples requires the possibility of using smaller quantities of samples, approximately 10 times lower than that of a rat, due to their smaller size. Additionally, studies regarding neurodegeneration often include behavioral assessments, necessitating individual quantification of mycotoxin levels in each mouse to maintain traceability. Related to this, a higher number of animals are commonly used in these studies to be able to reach conclusions from behavioral studies, so achieving rapid OTA quantification is also essential to process the high number of samples efficiently. Furthermore, specific validation of the method for each species is strongly recommended by the reference Guidelines, both from the Food and Drug Administration (FDA) [21] and the European Medicines Agency (EMA) [22], to ensure the reliability of the data generated. 

In this study, we validated a high-performance liquid chromatography–fluorescence detection (HPLC-FLD) analytical procedure suitable for the quantitative analysis of OTA in plasma, brain, kidney, liver, and intestine tissue in individual mice, making it possible to acknowledge interindividual variability. Guidelines from both the FDA [21] and the EMA [22] were consulted to ensure comprehensive validation of the method. Moreover, its effectiveness has been demonstrated via its successful application to the analysis of samples obtained after a 28-day OTA repeated intraperitoneal dose study in mice. 

## 2. Results

### 2.1. Method Validation

#### 2.1.1. Selectivity

**Figure 1:**
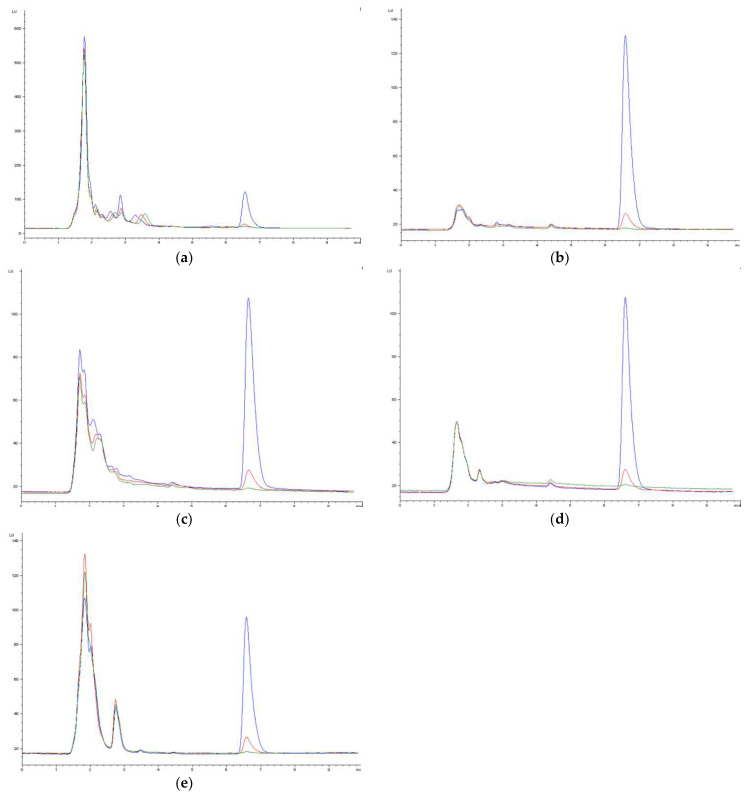
Chromatograms of blank samples of plasma (**a**), brain (**b**), kidney (**c**), intestine tissue (**d**), and liver (**e**) spiked with OTA. Green line = blank samples spiked with OTA as in the recovery study (2.35 ng/mL in plasma and 9.4 ng/g in brain, kidney, intestine tissue, and liver); red line = blank samples spiked with OTA as in the recovery study (22.83 ng/mL in plasma and 91.32 ng/g in brain, kidney, intestine tissue and liver); blue line = blank samples spiked with OTA as in the recovery study (228.33 ng/mL in plasma and 913.32 ng/g in brain, kidney, intestine tissue and liver).

The method was selective in all the matrices. The chromatograms of blank samples revealed no interfering signals at the retention time of OTA (6.7 min), and the retention time of OTA matches in both fortified plasma or tissues and real samples, as can be seen in Figure 1.

#### 2.1.2. Linearity

The calibration curves made on three different days for each one of the two concentration ranges (2.35–22.83 ng/mL and 22.83–228.33 ng/mL) showed a good linear relationship between peak areas and OTA concentrations. All the requirements for linearity have been met in all the cases (see Table S1 in the Supplementary Materials). Since the triplicated calibration curves for each range did not differ significantly, a global calibration curve for the 2.35–22.83 ng/mL range and another one for the 22.83–228.33 ng/mL range were made with the average of the triplicates. The results of the linearity study on the global calibration curves are presented in Table 1. The LLOQ for OTA was determined as the lowest calibration standard (2.35 ng/mL) as it showed acceptable accuracy and precision. Therefore, considering the dilution factors, the respective values of LLOQ in the different matrices are 2.35 ng/mL in plasma and 9.4 ng/g in brain, kidney, intestine tissue, and liver.

#### 2.1.3. Precision and Accuracy

Precision and accuracy of linearity showed adequate values according to FDA and EMA guidelines (Table 2). Raw data are available in the Supplementary Materials (Table S2).

#### 2.1.4. Recovery

The recovery values obtained for each matrix are summarized in Table 3. More data are provided in the Supplementary Materials (Table S3). Recovery was very efficient in all the matrices (74.8% for plasma, 79.7% for brain, 87.6% for kidney, 80.2% for intestine tissue and 76.2% for liver). Furthermore, the %CVs obtained in within- and between-day experiments were below 12% in each case, thus demonstrating the precision of the methodology.

#### 2.1.5. Dilution Integrity

The dilution integrity of OTA from plasma samples that needed a dilution of the supernatant before the HPLC injection was also studied for the dilution factor selected (1/15). Accuracy and precision were both below 15% (respectively, 2.93% (mean RE%) and 5.54% (CV%)). This demonstrates that acidified ACN is not saturated when extracting high OTA doses and that the dilution of the supernatant does not affect the recovery. 

#### 2.1.6. Carry-Over Effect

There was no carry-over effect when measuring blank samples after high-concentration calibration standards. 

#### 2.1.7. Stability

OTA in processed plasma, brain, intestine, liver, and kidney samples was stable for at least 6 h in the autosampler tray (Table 4). Considering these results, all samples were analyzed immediately after extraction and in less than 6 h, in order to assure OTA quantification. Thus, a maximum of 24 vials were measured in one run, including samples, calibrators, and QCs. 

#### 2.1.8. Robustness

OTA retention time was very similar in both columns (6.7–6.9 min). When calibration standard areas obtained with column A were extrapolated with the global calibration curves obtained in column B and vice versa, the accuracy values obtained were acceptable according to guidelines (<15%). The results from the robustness study are summarized in Table 5. More data are available in the Supplementary Materials (Tables S4 and S5). 

### 2.2. Application to In Vivo Study

**Figure 2:**
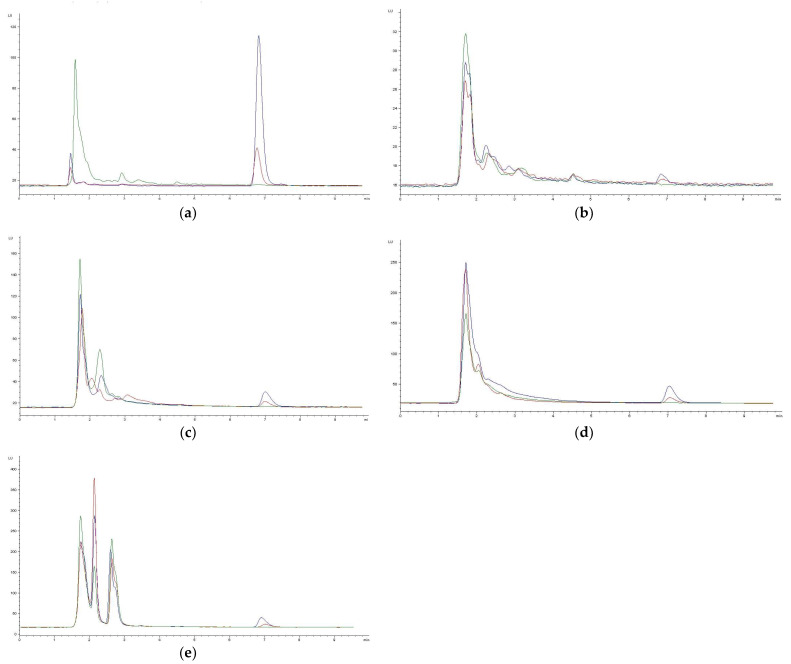
Representative chromatograms obtained in the analysis of samples from the in vivo study used in the application of the method in the different matrices: plasma (**a**), brain (**b**), kidney (**c**), intestine tissue (**d**), and liver (**e**). Green line = samples from control animals; red line = samples from treated animals (0.21 mg/kg bw); blue line = samples from treated animals (0.5 mg/kg bw). Plasma samples from treated animals (both 0.21 mg/kg bw and 0.5 mg/kg bw) were diluted 1/15.

This analytical procedure was applied to measure OTA in samples obtained after an OTA repeated intraperitoneal dose study. OTA levels in control samples were not detectable in any matrix, but they were found in all the matrices at both doses. Results are depicted in Table 6, and typical chromatograms obtained can be seen in Figure 2. The highest levels of OTA were found in plasma, followed by the liver. As explained above, all of the analytical results obtained were corrected by recovery.

## 3. Discussion 

The method presented in this work is the first one published, to the best of our knowledge, that allows OTA quantification in five different matrices (plasma, kidney, liver, brain, and intestine tissue) in mice by means of HPLC-FLD. Indeed, there is no HPLC-FLD method to quantify OTA in mice brains or intestines. There have been published several analytical methods for OTA determination in biological samples. However, most of them are developed for other species, mainly rats, and therefore, they are not applicable to mice tissues, as they need a high quantity of samples in comparison with the sample amount that can be extracted from a mouse. Considering a mouse weighs around 20–25 g and a rat approximately 200–250 g, the sample size required in a method validated for OTA quantification in mice would have to be 10 times lower than if it were validated for rats. 

Regarding methods used previously by other authors to quantify OTA in mice, except for the one published by Szöke et al. [23], they are all immunoassay methods, either enzyme immunoassays [24] or radioimmunoassays [25,26]. Immunological methods are typically less expensive but cannot distinguish between different ochratoxins, as reviewed by Meulenberg [27]. Moreover, the use of radiolabeled compounds has important disadvantages, as they present multiple health hazards and need specialized waste disposal. The method validated in this study allows to quantify OTA in five different tissues in each individual mouse by means of high-performance liquid chromatography coupled with a fluorescence detector. HPLC-FLD is one of the most sensitive, convenient, and widely used bioanalytical methods for OTA quantification in biological samples, as it is able to detect and distinguish between the various ochratoxin family members and metabolites. In fact, recently, Szöke et al. aimed to develop an immunoassay-based method that could be compared to HPLC-FLD (14). They do not present the full validation data for the chromatographic method used; however, they concluded that HPLC-FLD gave, indeed, the most reliable measurement at the lowest levels of OTA. 

Apart from the high specificity, one important advantage of the present method is that only 50 μL of plasma and 12.5 mg of tissue are enough to obtain results in a wide range of concentrations. The low sample mass needed is extremely relevant in toxicology and toxicokinetic studies, as well as when studying neurodegeneration. In this last situation, the mice’s brains must often be dissected to analyze different structures separately, and therefore, the sample mass available for HPLC analysis can be very low (15–20 mg), being this one of the critical factors. Many authors do not specify the tissue mass needed for the analysis [23], or they propose to use pooled tissues [24], which entails increasing the number of animals per group and losing the interindividual variability data. Additionally, working with such small volumes also accelerates the process, as the drying process is less time consuming (around 30–40 min), being able to process samples in less than 3 h.

Regarding the range of concentrations, we proved the linearity of the method between 2.35 and 228.33 ng/mL, defining 2.35 ng/mL as the LLOQ for plasma and 9.4 ng/g as the LLOQ for brain, kidney, liver, and intestine. In their publication, Szöke et al. [23] determined the linearity in a higher range of concentrations (between 4.0 and 403.8 ng/mL), although they defined the LLOQ as 2.4 ng/mL based on signal-to-noise ratio without checking the linearity, precision, or accuracy. Thus, the present method achieved a similar LLOQ while simultaneously demonstrating dilution integrity for expected high-concentration samples. This capability enabled the quantification of OTA across a broader concentration range, even after administering low doses of the mycotoxin. 

The method presented allows OTA quantification in five different matrices (plasma, kidney, liver, brain, and intestine). Tissue analysis requires an additional step in sample preparation to turn biological samples into a liquid form. To this end, there are several techniques available, such as homogenization, digestion, or sonication. Homogenization is the most popular one [23]; however, the risk of cross-contamination is high, and the homogenizer should be thoroughly rinsed after each sample to avoid it. In this study, the validated method includes bead beating as the cell-disrupting procedure. Bead beating is a cell disruption technique that can be used to obtain DNA, RNA, proteins, metabolites, and small molecules from diverse samples like animal and human tissues, bacteria, plants, etc. [28]. This technique has become more popular lately, as it minimizes the risk of cross-contamination compared to traditional techniques while also simplifying, optimizing, and accelerating the sample preparation process. In this way, tissue samples are processed in individual tubes that contain grinding beads, and 40 s is enough to obtain the homogenates. Simultaneously, three samples can be homogenized with the model used, although the number can be up to 24 samples at the same time with a bigger apparatus. It is the first time, to our knowledge, that the bead beating technique has been used in mice tissue samples for OTA extraction, proving to be a useful and easy process.

Once tissues are homogenized, the extraction method is common for four out of the five matrices (plasma, kidney, brain, and intestine tissue) using a three-fold volume of ACN acidified with 0.4% formic acid. ACN is one of the most common organic precipitants for the pretreatment of tissue samples due to its strong precipitating ability [29]. However, the extraction of OTA has been demonstrated to be pH dependent, as at higher pHs (above pH 5.0), the deprotonation of the carboxyl and/or the phenolic hydroxyl group(s) can lower the efficacy of the extraction [30]. Thus, acidifying ACN with 0.4% formic acid allows the most effective extraction of OTA. The selection of the solvent was also made according to previous studies regarding the extraction of mycotoxins, which demonstrated that acidified ACN was the best solvent for the matter [31]. For protein precipitation, at least two volumes of organic solvent should be added to each unit weight of tissue; the more added, the more dilution factor but also, the more thorough the precipitation will be [29]. With the method developed, a three-fold volume is used to ensure protein precipitation as then the supernatant is evaporated, and no dilution is made in this step. In the case of the liver, ice-cold absolute ethanol and trichloroacetic acid 20% were used as ACN acidified with formic acid did not clean enough the samples. Amongst the options available to further clean the samples, the use of a salt like sodium acetate was discarded as it has been shown to reduce recovery values [31]. In the same line, cartridges could not be used due to the small volumes available. Ethanol is also widely used as a precipitation agent and is less toxic than other organic solvents such as chloroform or acetic acid. Again, the addition of a strong acid as trichloroacetic acid 20% lowers the pH of the extraction solvent making the extraction process more effective. However, it has to be noted that TCA is considered to be hazardous, and the Threshold Limit Value (TLV) established by the International Labour Organization (ILO) is much lower than those admitted for acetonitrile, formic acid and ethanol (0.5 ppm for TCA, 20 ppm for ACN, 5 ppm for formic acid and 1000 for ethanol) [32]. Therefore, to lower the risks, the extraction process was carried out with acidified ACN when possible. In both cases, the procedure is simple, fast, and economical because only one solvent step is needed, and the use of immunoaffinity columns is not necessary. Five minutes of vortexing was enough time with both extraction solvents to extract OTA from complex matrices such as the kidney, liver, plasma, intestines, and brain.

The recovery values have been studied and are presented for each matrix, which is important considering the differences in composition between the matrices that affect OTA binding and releasing. Recovery values are very efficient in all the matrices, ranging between 74.8 and 87.6%, with CV values below 15%. This good reproducibility validates the procedure of spiking blank samples and demonstrates the precision of the analytical procedure. Szöke et al. [23] obtained a higher recovery value, but they do not specify whether it is for plasma or for tissue samples nor present the within- and between-day variability. However, from their study it is clear that recovery values obtained from HPLC analysis are higher than those obtained from ELISA-based immunoassay or from flow cytometry measurements. These findings reinforce the idea of HPLC being an adequate technique to quantify OTA in mice tissues.

The fact that the method presented uses the same calibration curves and chromatographic conditions for all five matrices makes it easier to carry out the experimental work. The mobile phase used is also easy to prepare, as there is no need to adjust the pH. The LLOQ achieved in all the matrices was adequate for toxicological studies, where high doses are administered to small animals, but also could be applied to further studies in the neurodegeneration field, in which lower doses are administered for longer periods of time, as proven in the application of the method. 

OTA concentrations were found to be similar in the kidney and liver, with slightly higher levels detected in the liver. While this finding may appear contradictory to OTA’s primary nephrotoxic effect, it aligns with previous studies in rodents that reported similar or even higher OTA levels in the liver under various experimental conditions and administration routes [23,33,34,35,36,37,38,39]. In this regard, some of these studies have demonstrated that, despite similar accumulation in both target and non-target tissues, only the kidney exhibited biochemical and histopathological changes [36,37]. Moreover, the results also show OTA accumulation in the small intestine at levels similar to those in the kidney and liver and penetration via the blood–brain barrier, with concentrations in the brain falling between the LOD and the LLOQ. This is in agreement with Wang et al., who in 2020 reported similar biodistribution of OTA upon intravenous administration [25]. It is important to note that in this method tissue levels and not intestine content levels were measured, as in the latter, levels of OTA can be substantially higher [25]. However, considering that OTA-induced toxicity is not only driven by tissue distribution and kinetics but also by organ-specific toxicodynamics, the necessity for further delving into its possible role in neurodegenerative diseases is underscored.

## 4. Conclusions

In conclusion, a highly sensitive, rapid, and simple HPLC-FLD method for OTA determination in mice tissues (kidney, liver, brain, and intestine), as well as plasma samples, was developed and validated for selectivity, linearity, accuracy, precision, recovery, dilution integrity, carry-over effect, stability, and robustness. The applicability of the assay was evaluated in repeated low-dose OTA study in Balb/c mice.

The method described allows for the quantification of OTA in each individual needing a very low sample mass with good recovery values. In addition, it has been validated for novel and less common tissues in OTA-related research, such as the intestine and brain, which could, however, be essential for delving into its potential neurodegenerative effects. Moreover, the fact that immunoaffinity columns are not needed makes this also a simple, fast, and economical method. Following the FDA and EMA guidelines, it has been demonstrated that all the validation criteria were met; thus, the method has adequate characteristics to assure reliable results.

## 5. Material and Methods

### 5.1. Reagents

All the reagents used for the HPLC analysis were of LC-gradient grade. Acetonitrile (ACN) and formic acid were purchased from Merck (Darmstadt, Germany), whereas absolute ethanol UV–IR-HPLC and trichloroacetic acid 20% (*w*/*v*) (TCA) were both obtained from Panreac (Barcelona, Spain). Water used throughout the analysis was purified with a Milli-Q System (Millipore, Bedford, MA, USA). 

For the tissue homogenates, sodium phosphate buffer (0.05 M, pH 6.50) was prepared by adding 6.90 g of NaH_2_PO_4_·H_2_O (Merck, Darmstadt, Germany) to 900 mL of distilled water. The pH of the dissolution was adjusted with NaOH 3 M, and the volume was adjusted to one liter. The buffer was kept at 4 °C until use. 

OTA was obtained in powder from Sigma-Aldrich (Steinheim, Germany) (REF O1877, lot 0000149830). For animals’ administration, OTA was dissolved in 0.1 M NaHCO_3_ (sodium bicarbonate powder, Sigma-Aldrich, Steinheim, Germany), adjusted to pH 7.4 with HCl, and kept at −20 °C until use. To prepare the standard solutions, OTA was dissolved in methanol 99.9% (Panreac, Barcelona, Spain) and kept at −20 °C until use.

### 5.2. Application of the Method: Animals and Samples Collection

#### 5.2.1. Animals and Experimental Design

The animals used were eight- to nine-week-old male Balb/cByJ (ref. 627) mice purchased from Charles River. On the day of arrival, animals were weighted and distributed randomly into individual polycarbonate cages with stainless steel covers, with a maximum of six mice per cage. Mice were maintained in constant environmental conditions of humidity (55 ± 10%) and temperature (22 ± 2 °C) on a 12 h light/dark cycle and allowed ad libitum access to standard pellet diet (Special Diet Service, Essex, UK) and normal tap water.

For the study involved in the application of the method, animals were distributed into 3 groups (n = 10 per group) and, after one week of acclimatization, received repeated OTA administrations (0.21 or 0.5 mg/kg) or vehicle (NaHCO_3_) daily for 28 days, intraperitoneally. For blank samples, male Balb/cByJ (ref. 627) (n = 10) were purchased, left for the acclimatization period, and sacrificed without receiving any administrations. 

These experiments were approved by the Ethics Committee on Animal Experimentation of the Universidad de Navarra (CEEA 049-19), and they were conducted according to the National Institute of Health (NIH) Guide for the Care and Use of Laboratory Animals.

#### 5.2.2. Plasma and Samples Collection 

Animals were sacrificed 24 h after the last OTA administration. Blood was obtained at sacrifice from cardiac puncture and collected in Sarstedt (Nümbrecht, Germany) Multivette^®^ 600 EDTA K3 tubes for then to be centrifuged at 2000× *g* for 10 min. The obtained plasma was stored at −20 °C until HPLC analysis. The brain, liver, kidney, and small intestine were rapidly removed from the animals and washed with saline buffer until the external blood was removed. Intestines were also washed internally. Organs were carefully dried using filter paper and weighed; then, the brain was dissected, and the rest of the organs were carved. During the necropsies, all the dissection material was cleaned with saline buffer and rinsed with ethanol after each animal to prevent sample contamination.

For other purposes, the brain was dissected, and the different structures were collected as needed. After dissection, the remaining portion of the right hemisphere was promptly frozen in liquid nitrogen for OTA quantification. The kidneys, liver, and intestine were carved according to established laboratory protocols: the kidney was transversely cut to include both cortex and medulla, one lobule of the liver was longitudinally cut, and the intestine was transversely cut. Thereafter, smaller portions of these tissue pieces were immediately frozen in liquid nitrogen and kept at −80 °C until analysis. The weight of these tissue pieces varied between organs, ranging from 15 to 20 mg for the brain and 18 to 40 mg for the other tissues. Blank samples for method validation were obtained from non-treated animals and processed as described above. 

### 5.3. Apparatus and Chromatographic Conditions

The analytical method was based on the one validated for rat plasma, kidney, and liver by our group [40], with some modifications in order to adapt the sample treatment to a different animal species and to new tissues (brain and intestine), reducing the sample mass needed, and also, to simplify both: chromatographic separation conditions (mobile phase) and sample preparation procedure. OTA was quantified by HPLC-FLD in an 1100 series LC (Agilent Technologies, Waldbronn, Germany) with a fluorescence detector (λ excitation 225 nm and λ emission 461 nm). The chromatographic system was equipped with a Tracer Extrasil ODS column (25 cm × 0.4 cm, 5μm particle size) from Teknokroma (Spain) preceded by a (4 mm i.d.) Tracer Extrasil ODS2 safeguard column and working at 40 °C. The mobile phase was a mixture of acetonitrile and an aqueous solution of formic acid (0.4%) (50:50) in isocratic conditions. The aqueous phase was filtered through a 0.45 μm nylon membrane filter (Teknokroma, Barcelona, Spain). The injection volume was 20 μL, and the flow rate was 1 mL/min. The retention time for OTA under these conditions was 6.7 min, and the total analysis time was 10 min.

### 5.4. Preparation of Stock and Working Solutions

The stock solution of OTA (1 mg/mL) was prepared by dissolving 1 mg of OTA in methanol, and its concentration was verified by spectrophotometry at 333 nm (MW = 403.8; ε = 5500 M^−1^ cm^−1^). OTA working standard solutions were prepared by diluting the stock solution with methanol and stored at −20 °C until use. OTA stability was previously confirmed in these conditions [41]. Thirty minutes before using them, an aliquot of each standard was tempered in darkness. 

### 5.5. Preparation of Calibration and Quality Control (QC) Samples 

The calibrators used in the validation of the method were prepared in 1.5 mL Eppendorffs by evaporating 50 μL of the corresponding working standard solution and dissolving them in 50 μL of the mobile phase. 

QC samples were prepared similarly to calibrators at low, medium, and high concentration levels (2.35 ng/mL, 22.83 ng/mL, and 228.33 ng/mL) and were included in every analytical run while the analysis of study samples, according to the recommendations in FDA end EMA guidelines [21,22].

During sample analysis, according to the guidelines, each analytical run contained the three QC levels and at least two replicates per QC level. In total, at least six QCs were used in each analytical run, or QCs were enough to accomplish 5% of the total number of the analyzed samples, whichever number was greater. The preparation of all calibrators and QC samples was just before the analysis. 

### 5.6. Sample Treatment

#### 5.6.1. Homogenization of Solid Tissues

In the case of solid tissues (brain, liver, kidney, and intestine), frozen samples were thawed, weighed, and mixed with a sodium dihydrogen phosphate-buffered solution at pH 6.5 (4 μL per mg of tissue, dilution factor of kidney, liver, and intestines 1/4). After that, the mixture was homogenized with a bead beater (BeadBug™ 3 Position Bead Homogenizer, Gentaur, Kampenhout, Belgium) using 2 mL screw cap microtubes and 3 mm glass beads. Homogenates were then transferred to another Eppendorf in order to be frozen without the presence of the beads at −80 °C. Homogenates were frozen for at least 24 h hours until the extraction was carried out. 

#### 5.6.2. OTA Extraction

**Plasma, brain, kidney, and intestine.** For OTA quantification in plasma, brain, kidney, and intestine tissue homogenates, frozen samples were kept at room temperature in darkness for 30 min before the extraction process. Following that, 50 μL of the sample was mixed with 150 μL of ACN acidified with formic acid (0.4%) for protein precipitation and OTA release. After vortexing for 5 min, the sample was centrifuged at 12,000× *g* for 10 min at room temperature. Then, 150 μL of the supernatant were dried in an evaporator (GeneVac, SP Scientific, Ipswich, England) under vacuum at 60 °C and reconstituted with 50 μL of mobile phase (no dilution factor for plasma, dilution factor of kidney, liver, and intestines 1/4). For plasma samples with an expected high OTA concentration, only 10 μL of the supernatant was evaporated before being reconstituted with 50 μL of mobile phase (dilution factor 1/15).

**Liver.** For OTA extraction in the liver, homogenates were tempered in darkness for 30 min at room temperature. After that, 50 μL of the sample was mixed with 120 μL of ice-cold absolute ethanol and 15 μL of TCA 20%. Then, samples were vortexed for 5 min, centrifuged at 12,000× *g* for 10 min at room temperature and treated further as previously described for plasma, brain, kidney, and intestine tissue samples.

### 5.7. Validation of the Method

Following the FDA end EMA guidelines [21,22], the analytical method was validated according to the following parameters: selectivity, linearity, accuracy, and precision (within and between days), recovery, dilution integrity, carry-over effect, stability, and robustness. The acceptance criteria, based on the guidelines, are summarized in Table 7. 

#### 5.7.1. Selectivity 

The ability of the method to distinguish OTA from other endogenous components present in the samples was evaluated for the five matrices individually. The selectivity of the method was assessed by measuring and comparing blank plasma or tissues from 6 different individuals (not treated with any substance), with blank plasma or tissues spiked with OTA and with samples from OTA-treated mice.

#### 5.7.2. Linearity (Calibration Curves) and LLOQ

The linearity was tested with OTA calibrators prepared as described above in 5.5. A calibration curve was made in triplicate on three different days in each one of the following ranges: 2.35–22.83 ng/mL and 22.83–228.33 ng/mL. Each one of the ranges included six points. With regard to linearity, the following criteria were considered: correlation coefficient (*r*^2^ > 0.99), coefficient of variation (CV) between response factors (<5%), slope interval not having to include zero (*p* = 95%), and intercept interval having to include zero (*p* = 95%). Also, the back-calculated concentrations of the calibration standards should be within 15% of the nominal value (20% at LLOQ).

The lower limit of quantification (LLOQ) was established as the lowest OTA concentration inside the linear range that could be quantified with acceptable precision and accuracy (±20%).

#### 5.7.3. Precision and Accuracy

Within- and between-day precision and accuracy of the linearity were studied by analyzing three replicate calibrations standards at low, intermediate, and high concentrations (2.35, 22.83, and 228.33 ng/mL) on 1 day (within days) and on 3 different days (between days). The accuracy was calculated as the relative error (RE%) of back-calculated concentrations with respect to the nominal value. The precision, defined as the closeness of repeated individual measures of the analyte, was expressed as CV% between the different replicates (within days) and runs (between days). Criteria for precision and accuracy were, respectively, CV (%) and RE (%) of less than 15% (20% for LLOQ).

#### 5.7.4. Recovery

Due to the difficulty of obtaining these blank matrices in quantity enough to prepare all the matrix-matched calibrators and QCs needed, linearity has been studied using OTA solutions. For this reason, the recovery of OTA when the extraction procedure was applied to the samples has been assessed. Recovery has been studied for each one of the matrices, fortifying blank samples of each matrix with known OTA concentrations (2.35, 22.83, and 228.33 ng/mL). The spiking process was as follows: 50 μL of the corresponding working OTA solution was poured into an Eppendorf and evaporated under a vacuum at 60 °C. Then, 2.5 μL of ACN acidified with formic acid (0.4%) was added, and after vortexing for 2 min, 50 μL of the blank sample (plasma or tissue homogenates) were added and vortexed for 1 min more. The addition of this small volume of acidified ACN was made to ensure that plasma or homogenates dissolved OTA residue completely following evaporation. The mixture was left in darkness at room temperature to stand for 10 min before starting the procedure of sample treatment as previously described. The OTA recovery value (%) in all the matrices was calculated by dividing the experimental OTA concentration obtained in the spiked samples by the nominal OTA level. The precision of this process was studied by carrying out the recovery experiment for each matrix at three concentration levels, by triplicate, and on 3 different days so precision could be assessed in intermediate conditions. The criteria for precision was CV (%) of less than 15% (20% for LLOQ). The obtained mean recovery values for each one of the matrices were used in the correction of the levels obtained using the calibration curves.

#### 5.7.5. Dilution Integrity

Some plasma samples were expected to have a high OTA concentration, so they were diluted. For this reason, dilution integrity was proven. For that aim, the dilution factor chosen (1/15) was used in a blank plasma sample spiked with an OTA concentration above the upper limit of quantification (ULOQ) (1174.69 ng/mL)**.** Five determinations were made in one day, and the acceptance criteria were accuracy and precision (respectively RE% and CV%) of less than 15%.

#### 5.7.6. Carry-Over Effect

The carry-over effect was investigated by measuring blank samples after calibration standards at the ULOQ five times. 

#### 5.7.7. Stability

OTA stability in frozen tissue homogenates was not assessed in this study, as previous data using rat tissues and the same homogenization buffer showed that OTA is stable at least 10 months after being homogenized and frozen [40]. OTA stability in the HPLC autosampler tray was evaluated by analyzing OTA spiked samples of each matrix at LLOQ and at ULOQ just after preparation (0 h) and at 2, 6, and 12 h in the autosampler tray. The stability of OTA in the analysis solution was accepted if RE% of the OTA concentration obtained is between ±15% of the one just after sample preparation. Additionally, an OTA concentration versus time regression analysis was performed; stability was confirmed if the slope was not statistically different from 0 (*p* = 95%).

#### 5.7.8. Robustness

Finally, robustness was assessed by studying the influence of different batches of the chromatographic column. Linearity on two different Tracer Extrasil ODS2 columns was studied by analyzing calibrators in each one of them. Linearity was studied as presented before in each one of the columns and for both ranges studied (2.35–22.83 ng/mL and 22.83–228.33 ng/mL), and a global calibration curve was obtained in each one of the ranges and columns. After that, the peak areas of OTA obtained in one of the columns for calibrators at the low, medium, and high levels (2.35, 22.83, and 228.33 ng/mL) were extrapolated and quantified with the global calibration curves obtained in the other column. Robustness was assessed if precision and accuracy for the calibrators were less than 15% in each one of the columns.

### 5.8. Acceptance Criteria of an Analytical Run 

According to the guidelines [21,22], the run was considered acceptable if at least 67% of QCs were within ± 15% of the nominal values and 50% or more of QCs per level were within ±15% of their nominal concentrations.

## Figures and Tables

**Table 1:** Global calibration curves obtained in the linearity study in the following ranges: 2.35–22.83 ng/mL and 22.83–228.33 ng/mL. Eighteen data points were used for each calibration range.

	Range 2.35–22.83 ng/mL	Range 22.83–228.33 ng/mL
Curve equation ^a^	y = 9.19x + 0.533	y = 8.50x + 15.33
r^2^	0.997	0.999
Slope limits (*p* = 95%)	8.55; 9.83	8.19; 8.81
Intercept limits (*p* = 95%)	−6.96; 8.03	−20.61; 51.27
CV ^b^ of response factors (%)	3.72	3.47
Back-calculated RE ^c^ (%)	<5.5	<5.2

^a^ y: peak area, x: concentration of OTA (ng/mL for plasma or ng/g for tissues).^b^ Coefficient of variation. ^c^ Relative error.

**Table 2:** Results of the precision and accuracy study. The precision within days was studied by analyzing some calibrators (2.35, 22.83, 228.33 ng/mL) in triplicate each day. The precision between days was assured by analyzing calibrators of these levels in three different days.

	Within-Day Variability (n = 3)			Between-Day Variability (n = 9)		
C_nominal_ (ng/mL)	C_measured_ (ng/mL)	CV ^a^	A ^b^	C_measured_ (ng/mL)	CV ^a^	A ^b^
Range 2.35–22.83 ng/mL ^c,d^						
2.35	2.21	4.08	2.09	2.29	3.55	2.55
22.83	21.77	2.09	4.64	21.63	4.95	5.25
Range 22.83–228.33 ng/mL ^e,f^						
22.83	22.43	2.13	1.77	22.28	4.95	2.42
228.33	235.82	0.49	3.28	226.25	4.99	0.91

^a^ Coefficient of variation (%). ^b^ Accuracy (RE%). ^c^ Equivalent range in plasma: 2.35–22.83 ng/mL (no dilution factor). ^d^ Equivalent range in kidney, liver, brain, and intestine tissue: 9.4–91.32 ng/g (dilution factor: 4). ^e^ Equivalent range in plasma: 342.45–3424.95 ng/mL (dilution factor: 15). ^f^ Equivalent range in kidney, liver, brain, and intestine tissue: 91.32–913.32 ng/g (dilution factor: 4).

**Table 3:** Results of the recovery study. The repeatability of the process was studied by carrying out the complete recovery experiment for each matrix on 1 day (within days) and on 3 different days (between days).

		Global Recovery (%)	CV ^a^ (%)
Plasma	Within-day	75.2 (n = 9)	6.8 (n = 9)
	Between day	74.8 (n = 27)	7.5 (n = 27)
Brain	Within-day	76.9 (n = 9)	12.8 (n = 9)
	Between day	79.7 (n = 27)	11.2 (n = 27)
Kidney	Within-day	88.8 (n = 9)	2.9 (n = 9)
	Between day	87.6 (n = 27)	4.5 (n = 27)
Intestine	Within-day	80.4 (n = 9)	11.8 (n = 9)
	Between day	80.2 (n = 27)	11.6 (n = 27)
Liver	Within-day	78.9 (n = 9)	5.5 (n = 9)
	Between day	76.2 (n = 27)	5.8 (n = 27)

^a^ Coefficient of variation.

**Table 4:** Results of stability study. Time evolution of OTA concentrations in fortified blank samples at low and high concentrations.

	Plasma		Brain		Kidney		Intestine		Liver	
Time (h)	C_measured_ (ng/mL)	A ^a^ (%)	C_measured_ (ng/g)	A ^a^ (%)	C_measured_ (ng/g)	A ^a^ (%)	C_measured_ (ng/g)	A ^a^ (%)	C_measured_ (ng/g)	A ^a^ (%)
C_nominal_ 2.35 ng/mL										
0	2.44	3.99	2.14	9.10	2.43	3.44	2.24	4.74	2.61	10.92
2	2.36	0.52	2.46	4.88	2.25	4.06	2.30	2.26	2.41	2.59
6	2.52	7.28	2.61	11.05	2.56	9.11	2.60	10.66	2.12	9.99
12	5.70	142.64	3.48	48.17	3.17	34.81	1.62	30.92	2.05	12.77
C_nominal_ 228.33 ng/mL										
0	237.81	4.15	208.54	8.67	202.43	11.34	213.38	6.55	213.77	6.38
2	230.46	0.93	229.75	0.62	197.13	13.67	206.65	9.50	216.87	5.02
6	247.86	8.55	256.83	12.48	228.21	0.05	227.34	0.43	195.83	14.23
12	462.34	102.49	357.30	56.48	279.62	22.46	232.49	1.82	202.84	11.16

^a^ Accuracy (RE%).

**Table 5:** Results of the robustness study. Precision and accuracy values of peak areas obtained in column A are quantified with calibration curves from column B. Precision between days (n = 9) is presented in the table.

	Areas from Column A Extrapolated with Calibration Curves Obtained in Column B			Areas from Column B Extrapolated with Calibration Curves Obtained in Column A		
C_nominal_ (ng/mL)	C_measured_ (ng/mL)	CV ^a^ (%)	A ^b^ (%)	C_measured_ (ng/mL)	CV ^a^ (%)	A ^b^ (%)
Range 2.35–22.83 ng/mL ^c,d^						
2.35	2.35	3.55	0.20	2.37	8.25	0.90
22.83	20.37	4.95	10.76	22.00	5.94	3.65
Range 22.83–228.33 ng/mL ^e,f^						
22.83	22.26	4.95	2.50	24.01	5.94	5.16
228.33	216.82	4.99	5.04	231.80	5.55	1.52

^a^ Coefficient of variation. ^b^ Accuracy (RE%). ^c^ Equivalent range in plasma: 2.35–22.83 ng/mL (no dilution factor). ^d^ Equivalent range in kidney, liver, brain, and intestine tissue: 9.4–91.32 ng/g (dilution factor: 4). ^e^ Equivalent range in plasma: 342.45–3424.95 ng/mL (dilution factor: 15). ^f^ Equivalent range in kidney, liver, brain, and intestine tissue: 91.32–913.32 ng/g (dilution factor: 4).

**Table 6:** Concentrations of OTA in plasma, brain, kidney, intestine, and liver in control (NaHCO_3_) and OTA-treated mice after 28 days of repeated intraperitoneal dose administration (0.21 and 0.5 mg/kg bw). Results are expressed as mean ± SD for each matrix and condition.

	OTA Concentration (ng/mL or ng/g)				
	Plasma	Brain	Kidney	Intestine	Liver
Control animals					
1	<LLOQ ^a^	<LLOQ	<LLOQ	<LLOQ	<LLOQ
2	<LLOQ	<LLOQ	<LLOQ	<LLOQ	<LLOQ
3	<LLOQ	<LLOQ	<LLOQ	<LLOQ	<LLOQ
4	<LLOQ	<LLOQ	<LLOQ	<LLOQ	<LLOQ
5	<LLOQ	<LLOQ	<LLOQ	<LLOQ	<LLOQ
Mean	<LLOQ	<LLOQ	<LLOQ	<LLOQ	<LLOQ
Treated animals (0.21 mg/kg bw)					
1	835.66	*3.05*	47.51	39.20	58.99
2	1093.83	*4.28*	56.58	56.78	69.65
3	836.21	*1.94*	55.41	52.84	78.98
4	787.23	*3.00*	48.18	35.80	69.35
5	849.31	*3.69*	78.81	54.15	60.96
Mean	880.45 ± 121.61	*3.19 ± 0.87*	51.14 ± 12.70	47.82 ± 9.47	67.59 ± 7.98
Treated animals (0.5 mg/kg bw)					
1	3858.11	13.19	163.70	296.86	244.00
2	1785.37	*5.72*	107.15	115.02	183.08
3	2312.75	*7.27*	106.93	149.33	163.17
4	3247.83	10.28	140.32	164.76	251.95
5	3061.26	9.90	172.70	104.34	173.59
Mean	2853.06 ± 812.59	*9.27 ± 2.89*	138.16 ± 30.77	166.06 ± 77.14	203.16 ± 41.61

^a^ Lower limit of quantification, Italics > LOD (limit of detection) < LLOQ.

**Table 7:** Summary of acceptance criteria used for the validation of the method (adapted from [21,22].)

Parameters	Criteria of Acceptance Based on EMA and FDA Criteria
Selectivity	Absence of interfering components is accepted where the response is not more than 20% of the analyte response at the LLOQ ^a^ for the analyte.
Linearity/calibration curve	At least 6 concentration levels. Back-calculated concentrations of the calibration standards should be within 15% of the nominal value (20% at LLOQ) for at least 75%.
Accuracy and precision	RE% ^b^ and CV% ^c^ (within runs and between runs): ±15% of nominal concentrations, except ± 20% at LLOQ.
Dilution integrity	RE% and CV%: ±15%.
Carry-over effect	Blank response after a calibrator at ULOQ ^d^ should not exceed 20% of the analyte response at LLOQ.
Stability	RE% at each level (LLOQ and ULOQ): ± 15%.
Robustness	Column batches (2).

^a^ LLOQ^:^ Lower limit of quantification. ^b^ RE%: relative error. ^c^ CV%: coefficient of variation. ^d^ ULOQ: upper limit of quantification.

## Data Availability

Data are contained within the article or the Supplementary Materials.

## Supplementary Materials

The following supporting information can be downloaded at https://www.mdpi.com/article/10.3390/toxins16050213/s1. Table S1: Raw data of the linearity study of column A; Table S2: Raw data from the precision and accuracy study; Table S3: Raw data from the recovery study; Table S4: Results of the robustness study; Table S5: Global calibration curves of column B.

## Abbreviations

ACN	Acetonitrile
CV	Coefficient of variation
EMA	European Medicines Agency
FDA	Food and Drug Administration
HPLC-FLD	High-performance liquid chromatography-fluorescence detection
IARC	International Agency for Research on Cancer
ILO	International Labour Organization
LLOQ	Lower limit of quantification
LOD	Limit of detection
NIH	National Institute of Health
OTA	Ochratoxin A
PD	Parkinson’s disease
QC	Quality control
RE	Relative error
TCA	Trichloroacetic acid
TLV	Threshold Limit Value
ULOQ	Upper limit of quantification
