# Supplementary material for: High-Performance Liquid Chromatography–Fluorescence Detection Method for Ochratoxin A Quantification in Small Mice Sample Volumes: Versatile Application across Diverse Matrices Relevant for Neurodegeneration Research
Source: Toxins (Basel). 2024 May 3;16(5):213. doi: 10.3390/toxins16050213 (PMC11125890; doi:10.3390/toxins16050213)
Supplement: Supplementary file 1 [file toxins-16-00213-s001.zip › toxins-2988112-supplementary.pdf]

Supplementary Materials: High-Performance Liquid Chromatography–Fluorescence Detection Method for Ochratoxin A Quantification in Small Mice Sample Volumes: Versatile Application across Diverse Matrices Relevant for Neurodegeneration Research

Elba Beraza, Maria Serrano-Civantos, Maria Izco, Lydia Alvarez-Erviti, Elena Gonzalez-Peñas and Ariane Vettorazzi

Table S1. Raw data of the linearity study of column A in the following ranges: 2.35 – 22.83 ng/mL and 22.83 – 228.33 ng/mL.

	Day 1	Day 2	Day 3
**Range 2.35 – 22.83 ng/mL**			
Curve equation[a]	y = 9.488x – 0.826	y = 9.256x – 0.995	y = 8.853x + 4.989
r^2	0.995	0.994	0.993
Slope limits (p=95%)	8.652; 10.32	8.131; 10.382	7.944; 9.762
Intercept limits (p=95%)	-10.63;8.976	-9.77; 7.78	-5.66; 15.65
CV[c] of response factors (%)	4.88	3.24	4.55
Back-calculated RE[b] (%)	< 7.5	< 4.2	< 7.4
**Range 22.83 – 228.33 ng/mL**			
Curve equation[a]	y = 8.639 x + 1.714	y = 8.585x – 5.356	y = 8.988x + 5.589
r^2	0.999	0.998	0.998
Slope limits (p=95%)	8.264; 9.015	7.976; 9.195	8.482; 9.493
Intercept limits (p=95%)	-41.84; 45.27	-82.59; 71.87	-53.10; 64.28
CV[c] of response factors (%)	4.03	4.14	4.39
Back-calculated RE[b] (%)	< 7.3	< 7.4	<7.2

[a] y: peak area, x: concentration of OTA (ng/mL for plasma or ng/g for tissues).

[b] Relative error.

[c] Coefficient of variation.

Table S2. Raw data from the precision and accuracy study. The precision within-day was studied by analyzing some calibrators (2.35, 22.83, 228.33 ng/mL) in triplicate each day. The precision between-day was assured analyzing calibrators of these levels in three different days.

	Day 1			Day 2			Day 3		
$C_{nominal}$ (ng/mL)	$C_{measured}$ (ng/mL)	CV[a]	A[b] (%)	$C_{measured}$ (ng/mL)	CV[a]	A[b] (%)	$C_{measured}$ (ng/mL)	CV[a]	A[b] (%)
**Range 2.35 – 22.83 ng/mL** [c,d]									
2.35	2.18	4.91	7.4	2.28	3.48	2.8	2.21		5.8
	2.40		2.1	2.35		0.1	2.29	4.08	2.7
	2.30		2.0	2.19		6.6	2.40		2.2
22.83	23.15	2.17	1.4	20.62	10.7	9.7	22.00		3.6
	22.83		0.0	20.44		10.5	22.06	2.09	3.4
	22.19		2.8	20.13		11.8	21.25		6.9
**Range 22.83 – 228.33 ng/mL** [e,f]									
22.83	23.88	2.22	4.6	21.22	1.25	7.1	22.67	2.13	0.7
	23.54		3.1	21.03		7.9	22.73		0.4
	22.86		0.1	20.70		9.3	21.87		4.2
228.33	211.50	1.83	7.4	224.42	2.40	1.7	235.50	0.49	3.1
	208.68		8.6	233.23		2.1	234.84		2.9
	216.34		5.3	234.65		2.8	237.11		3.8

[a] Coefficient of variation (%).

[b] Accuracy (RE%).

[c] Equivalent range in plasma: 2.35 – 22.83 ng/mL (no dilution factor)

[d] Equivalent range in kidney, liver, brain and intestine tissue: 9.4 – 91.32 ng/g (dilution factor: 4).

[e] Equivalent range in plasma: 342.45 – 3424.95 ng/mL (dilution factor: 15)

[f] Equivalent range in kidney, liver, brain and intestine tissue: 91.32 – 913.32 ng/g (dilution factor: 4).

Table S3. Raw data from the recovery study. The repeatability of the process was studied by carrying out the complete recovery experiment for each matrix on 1 day (within-day) and on 3 different days (between-day).

**$C_{nominal}$ (ng/mL)**		**2.35**			**22.83**			**228.33**		
		Day 1	Day 2	Day 3	Day 1	Day 2	Day 3	Day 1	Day 2	Day 3
	Measure 1	68.05	65.98	57.79	73.60	90.35	75.40	87.50	94.40	81.92
Brain	Measure 2	68.01	70.26	55.81	83.41	85.04	87.00	79.47	100.82	86.83
	Measure 3	61.98	71.60	69.16	81.68	93.72	77.34	88.85	107.49	88.55
	**Within-day recovery (n=3, first three dataset)**									
	Mean	66.01			79.56			85.27		
	CV[a] (%)	5.3			6.6			5.9		
	**Between-day recovery (n=9)**									
	Mean	65.40			83.06			90.65		
	CV[a] (%)	8.6			8.2			9.8		
	Measure 1	77.76	79.65	61.10	83.68	89.95	71.04	74.31	71.82	71.04
Liver	Measure 2	71.96	63.27	72.33	84.00	83.56	70.83	81.14	75.87	81.53
	Measure 3	73.76	74.91	68.33	81.97	83.14	74.19	81.06	80.75	74.96
	**Within-day recovery (n=3, first three dataset)**									
	Mean	74.49			83.22			78.84		
	CV[a] (%)	4.0			1.3			5.0		
	**Between-day recovery (n=9)**									
	Mean	71.45			80.26			76.94		
	CV[a] (%)	8.7			8.3			5.5		
	Measure 1	91.47	87.65	85.63	88.82	94.58	97.04	77.20	82.05	81.99
Kidney	Measure 2	86.37	91.66	69.85	92.41	93.46	88.66	90.21	87.67	84.31
	Measure 3	91.15	76.93	91.24	91.39	86.99	95.88	90.21	87.27	82.42
	**Within-day recovery (n=3, first three dataset)**									
	Mean	89.66			90.87			85.87		
	CV[a] (%)	3.2			2.0			8.7		
	**Between-day recovery (n=9)**									
	Mean	85.77			92.14			84.81		
	CV[a] (%)	8.9			3.8			5.1		
	Measure 1	75.05	61.84	73.80	81.33	77.07	71.09	93.43	85.87	97.99
Intestine	Measure 2	71.00	70.90	73.76	84.28	85.63	77.61	78.90	95.31	84.51
	Measure 3	65.89	77.76	68.65	77.01	86.54	78.77	96.60	77.82	96.47
	**Within-day recovery (n=3, first three dataset)**									
	Mean	70.64			80.87			89.64		
	CV[a] (%)	6.50			4.52			10.53		
	**Between-day recovery (n=9)**									
	Mean	70.96			79.93			89.66		
	CV[a] (%)	6.9			6.2			8.9		
	Measure 1	72.42	58.34	74.40	79.22	78.80	80.80	74.38	75.46	74.16
Plasma	Measure 2	73.02	64.88	72.98	80.41	80.39	79.76	72.38	74.56	74.78
	Measure 3	67.91	68.28	72.98	83.22	79.95	83.14	73.82	75.49	74.27
	**Within-day recovery (n=3, first three dataset)**									
	Mean	71.12			80.95			73.53		
	CV[a] (%)	3.9			2.5			1.4		
	**Between-day recovery (n=9)**									
	Mean	69.46			80.63			74.37		
	CV[a] (%)	7.5			1.9			1.3		

[a] Coefficient of variation.

Table S4. Results of the robustness study. Raw data of the linearity study of column B in the following ranges: 2.35 – 22.83 ng/mL and 22.83 – 228.33 ng/mL.

	**Day 1**	**Day 2**	**Day 3**
**Range 2.35 – 22.83 ng/mL**			
Curve equation[a]	y = 9.652x – 0.379	y = 9.348x + 0.434	y = 9.674x + 1.567
r^2	0.991	0.993	0.997
Slope limits (p=95%)	8.490; 10.815	8.365; 10.330	8.963; 10.386
Intercept limits (p=95%)	-14.01; 13.25	-11.09; 11.95	-6.78; 9.91
CV[c] of response factors (%)	6.13	6.55	4.20
Back-calculated RE[b] (%)	< 10.7	< 11.9	< 6.5
**Range 22.83 – 228.33 ng/mL**			
Curve equation[a]	y = 9.103 x + 1.949	y = 8.979x – 17.909	y = 9.240x – 7.715
r^2	0.999	0.998	0.998
Slope limits (p=95%)	8.264; 9.015	8.438; 9.520	8.776; 9.705
Intercept limits (p=95%)	-29.58; 33.47	-80.68; 44.87	-61.59; 46.16
CV[c] of response factors (%)	2.65	4.73	3.12
Back-calculated RE[b] (%)	< 3.5	< 11.3	<8.1

[a] y: peak area, x: concentration of OTA (ng/mL for plasma or ng/g for tissues).
[b] Relative error.
[c] Coefficient of variation.

Table S5. Global calibration curves of column B obtained as a result of the linearity study in the following ranges: 2.35 – 22.83 ng/mL and 22.83 – 228.33 ng/mL. Eighteen data points were used for each calibration range.

	**Range 2.35 – 22.83 ng/mL**	**Range 22.83 – 228.33 ng/mL**
Curve equation[a]	y = 9.54 x + 0.417	y = 9.08x – 2.33
r^2	0.995	0.999
Slope limits (p=95%)	8.72; 10.37	8.72; 9.44
Intercept limits (p=95%)	-9.22; 10.05	-44.25; 39.59
CV[b] of response factors (%)	4.59	4.82
Back-calculated ER (%)	< 7.55	< 5.0

[a] y: peak area, x: concentration of OTA (ng/mL for plasma or ng/g for tissues).
[b] Coefficient of variation.
